# Position Sensors for Industrial Applications Based on Electromagnetic Encoders

**DOI:** 10.3390/s21082738

**Published:** 2021-04-13

**Authors:** Ferran Paredes, Cristian Herrojo, Ferran Martín

**Affiliations:** CIMITEC, Departament d’Enginyeria Electrònica, Universitat Autònoma de Barcelona, 08193 Bellaterra, Spain; Cristian.Herrojo@uab.es (C.H.); Ferran.Martin@uab.es (F.M.)

**Keywords:** electromagnetic encoders, position sensors, microwave sensors, microstrip technology, motion control

## Abstract

Optical and magnetic linear/rotary encoders are well-known systems traditionally used in industry for the accurate measurement of linear/angular displacements and velocities. Recently, a different approach for the implementation of linear/rotary encoders has been proposed. Such an approach uses electromagnetic signals, and the working principle of these electromagnetic encoders is very similar to the one of optical encoders, i.e., pulse counting. Specifically, a transmission line based structure fed by a harmonic signal tuned to a certain frequency, the stator, is perturbed by encoder motion. Such encoder consists in a linear or circular chain (or chains) of inclusions (metallic, dielectric, or apertures) on a dielectric substrate, rigid or flexible, and made of different materials, including plastics, organic materials, rubber, etc. The harmonic signal is amplitude modulated by the encoder chain, and the envelope function contains the information relative to the position and velocity. The paper mainly focuses on linear encoders based on metallic and dielectric inclusions. Moreover, it is shown that synchronous electromagnetic encoders, able to provide the quasi-absolute position (plus the velocity and direction of motion in some cases), can be implemented. Several prototype examples are reviewed in the paper, including encoders implemented by means of additive process, such as 3D printed and screen-printed encoders.

## 1. Introduction

Optical linear and rotary encoder systems, providing either the incremental or the absolute position, are well stablished displacement and velocity sensors in industry [1,2,3,4,5,6]. Optical encoders are present in multiple applications involving motion control, including, elevators, pointing mechanisms, and servomotors, among others, and are key components in the automotive, aeronautic, and space industries. In terms of spatial or angular resolution, optical encoders are very competitive. For example, optical rotary encoders with thousands of pulses per revolution (PPR), the key figure of merit, are available in the market. Therefore, angular resolutions below 1 min of angle are possible. However, optical encoders are sensitive to the effects of harsh and hostile ambient, e.g., environments with pollution, contaminants, dirtiness, grease, etc. In addition, optical systems may be affected by other pernicious effects, such as those caused by radiation, extreme temperatures, etc., which can be found in certain scenarios.

Magnetic encoders are an alternative to optical encoders of special interest in applications subjected to the above-cited severe environments [7,8,9,10,11,12,13,14]. The reason is that magnetic components are tolerant against the effects caused by extreme temperatures, radiation or pollution/dirtiness. However, magnetic encoders use magnets or inductive elements, thereby representing an increase in cost or encoder design complexity. Recently, a new type of encoder systems operating at microwaves has been reported [15,16,17,18,19,20,21,22,23,24,25,26,27,28,29,30,31,32]. These encoders, designated as electromagnetic (or microwave) encoders, are the subject of this review paper. The stator (or reader) is a transmission line based structure, typically loaded with a resonant element (or with various resonant elements), fed by a harmonic signal (or with several harmonic signals). The movable element, the encoder, consists of a linear (for linear encoders) or circular (for rotary encoders) chain (or chains) of inclusions on a dielectric substrate, and such inclusions can be made of a metallic material (including conductive inks), of a dielectric material exhibiting permittivity contrast with the host substrate, or can be simple apertures drilled across the substrate. As the inclusions are in relative motion with regard to the sensitive element of the stator (the resonator), the transmission coefficient of the line at the frequency/ies of the feeding harmonic signal/s is modulated. The result is an amplitude modulated (AM) signal at the output port of the line containing the relevant information relative to encoder motion, i.e., position, velocity, motion direction, etc.

Several materials and inclusion types for the encoders, as well as different design strategies for the transmission line based stator, have been reported. This paper reviews and compares such approaches, with the focus on trying to highlight the advantages and drawbacks of the different implementations. The main contribution of this paper concerns the inclusion of the different reported approaches in a single manuscript, and a comparison on the basis of a table that reports encoder resolution and robustness. Paper organization is as follows. In Section 2, the working principle of the electromagnetic encoders is explained in detail. Section 3 focuses on the different encoder implementations, mainly oriented towards linear encoders. In Section 4, a comparative analysis of the different encoder types is carried out. Finally, the main conclusions are highlighted in Section 5.

## 2. Working Principle of Electromagnetic Encoders

**Figure 1:**
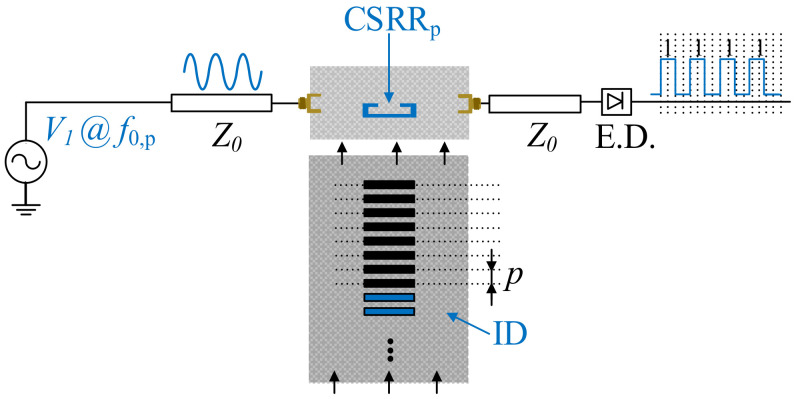
Sketch of the incremental electromagnetic encoder system.

The working principle of electromagnetic encoders is amplitude modulation (AM) of a harmonic signal injected to the stator, and caused by encoder motion [15,16,17,18,19,20,21,22,23,24,25,26,27,28,29,30,31,32,33]. Figure 1 depicts a sketch of this working principle, where (without loss of generality) the encoder consists of a single (linear) chain of linear inclusions. As the inclusions move above the sensitive part of the stator (reader), typically a resonant element loading a transmission line, the transmission coefficient of the line at the frequency of the feeding signal is modulated. Thus, the envelope function of the AM modulated signal at the output port of the line is expected to contain as many peaks, or dips, per unit time, as the number of crosses of the chain inclusions above the sensitive part of the stator.

In incremental-type encoders [15,16], a single chain suffices to determine the relative position of the encoder, as well as the instantaneous velocity. The latter is determined from the time lapse between adjacent peaks or dips in the envelope function of the AM modulated signal present at the output port of the line. Since the period of the chain is well known, it follows that the encoder velocity can be easily determined. On the other hand, the position is simply determined from the cumulative number of peaks or dips, recorded from a reference (REF) position. The sketch of Figure 1 corresponds, indeed, to an incremental electromagnetic encoder.

One limitation of incremental encoders is the impossibility to determine the position of the encoder in case of a system reset. For that purpose, absolute encoders are needed [28]. Optical absolute encoders are well known. In electromagnetic encoders, determining the absolute position is also possible. For that purpose, typically two different chains are needed (an exception is the encoder reported in [26]). One chain is codified by considering the presence (“1”) or absence (“0”) of functional inclusions at their predefined positions in the chain. The other chain contains all the inclusions, it acts as a clock providing the instants of times for reading the ID code of the other chain, and it gives the instantaneous velocity. Canonically, both chains can be distinguished by designating them with different names, e.g., clock (or velocity) chain and ID code (or position) chain.

Let us indicate the procedure to determine the absolute position of the encoder. For this purpose, we need to assign a unique code to the different positions of the encoder, discretized by the period, *p*, of the clock chain. If *L* is the total length of the encoder, the number of discrete positions is simply *L*/*p*, and the number of bits necessary to univocally differentiating the discrete position should satisfy

*N* ≥ log_2_*(L/p)* (1)

Thus, the bit inferred at a certain instant of time (dictated by the clock chain), plus the previous *N −* 1 bits, provide a unique *N*-bit sub-code that univocally identifies the encoder position. However, the complete ID code of the whole encoder chain, i.e., the bit sequence, should not be arbitrary, but chosen according to the De Bruijn sequence [34], which guarantees that any *N*-bit sub-code does not repeat for the total set of different positions of the encoder.

**Figure 2:**
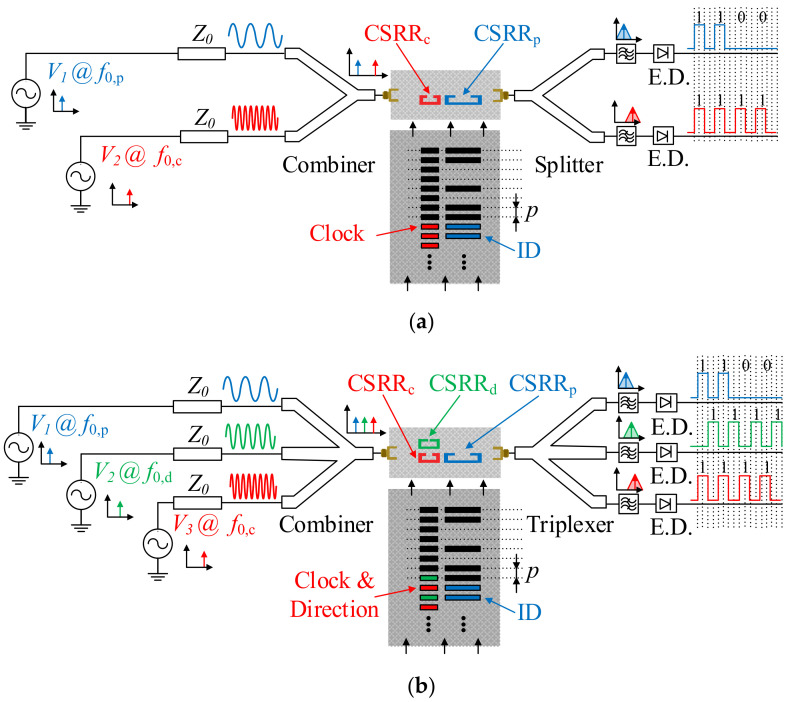
Sketch of the (**a**) quasi-absolute electromagnetic encoder system, and (**b**) quasi-absolute electromagnetic encoder system with motion direction detection capability.

In order to determine the encoder position, two different harmonic signals are needed. One of such signals should be AM modulated by the clock chain, whereas the position chain should modulate the other one. Obviously, the stator should contain two different sensitive elements, able to detect the presence or absence of inclusions for each chain. In practice, this is achieved by considering two independent resonators loading the host transmission line of the reader. The sketch of this absolute encoder is depicted in Figure 2a. Actually, these encoders should be designated as quasi-absolute encoders, since a number of bits *N* should be sequentially read (and therefore the encoder should move *N* positions) before an absolute position can be determined. The information relative to the velocity in these quasi-absolute encoders is also given by the time lapse between adjacent peaks or dips of the envelope function of the AM signal generated by the clock (velocity) chain. In certain applications it is also important to determine the motion direction. For that purpose, it is not necessary to consider an additional encoder chain, but to simply adding a third harmonic signal tuned to a different frequency. The idea behind the determination of motion direction is as simple as including an additional element (resonator) sensitive to the clock chain, but situated in a different position, so that a redundant clock signal is also inferred, but with a lag or led with regard to the main clock signal, depending on the motion direction. The sketch of this quasi-absolute encoder with motion-direction detection capability is depicted in Figure 2b.

It should be mentioned that for the quasi-absolute encoders of Figure 2, where at least two feeding harmonic signals should be injected to the transmission line of the stator, either a combiner or a switch managed by a microcontroller is required. With the combiner solution, the two or three harmonic signals are simultaneously injected to the transmission line. Each of these signals is AM modulated by encoder motion as described before. Thus, to collect the information relative to the absolute position, velocity and motion direction, a diplexer with filtering capability is needed. The envelope function can be inferred, e.g., by means of an envelope detector, preceded by an isolator, in order to avoid potential mismatching reflections caused by the diode, a highly nonlinear device. It is also possible to infer the envelope function of each channel by means of dedicated integrated circuits. If, alternatively to the combiner, the microcontroller/switching scheme is used, the harmonic feeding signals are injected sequentially to the input port of the transmission line of the stator. Nevertheless, the switching period is much shorter than the time lapse between adjacent peaks of the clock signal. Therefore, this approach provides also the information relative to the position, velocity, and motion direction of the encoder.

In the next section, several prototypes of electromagnetic encoder systems, including incremental and quasi-absolute implementations are discussed. Different inclusions, materials and fabrication processes of the encoders are included in the section. It is impossible to report all the implementations reported in the literature. Thus, we will focus mainly on linear encoders. Nevertheless, electromagnetic rotary encoders based on the reported principle will be succinctly referred to in the section, since these devices were the first electromagnetic encoders reported in the literature. It should be mentioned that the relevant performance parameter in encoders is the resolution, sometimes identified as the sensitivity in this type of devices. It is given by the number of pulses per revolution in incremental rotary encoders, by the number of pulses per unit length in incremental linear encoders, and by the number of bits in absolute encoders.

## 3. Prototype Examples of Electromagnetic Encoders

**Figure 3:**
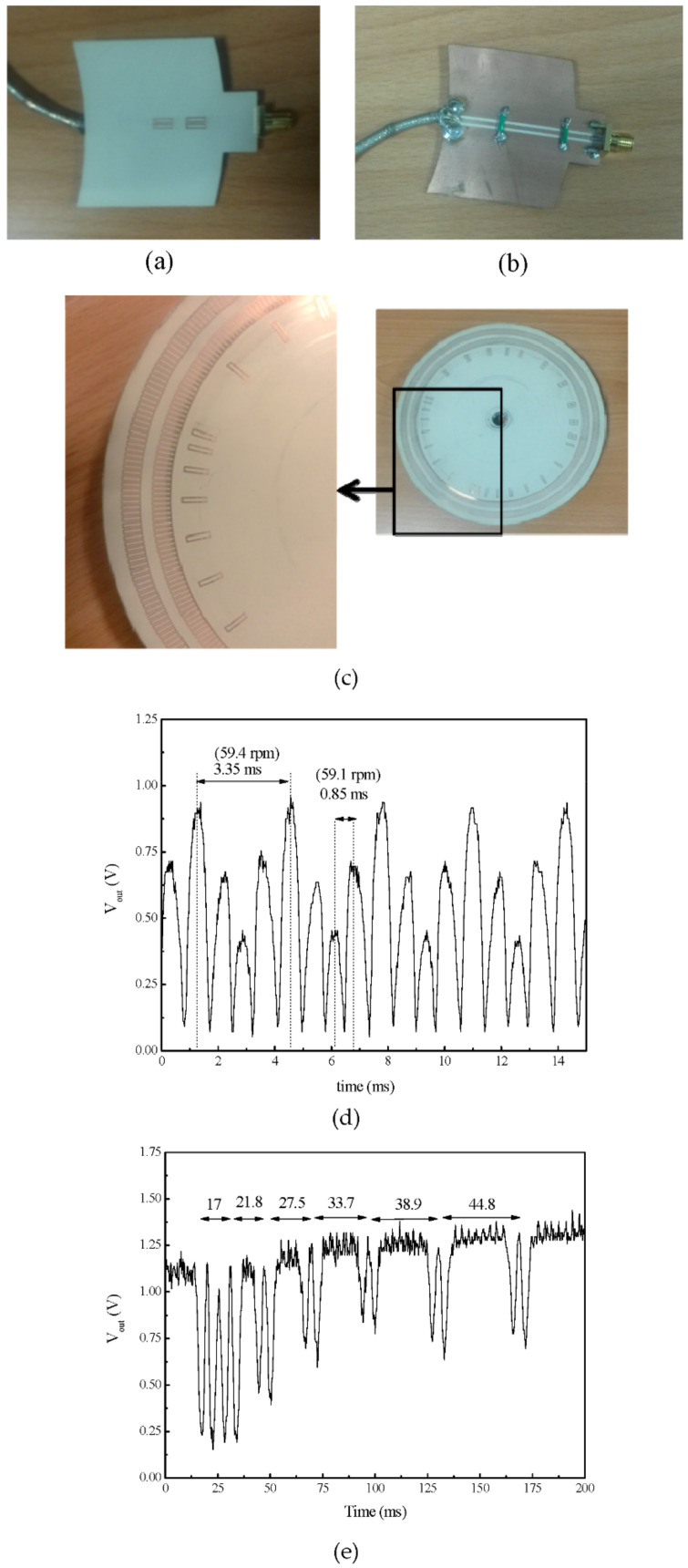
Example of an electromagnetic rotary encoder based on circular chains of split ring resonators. (**a**) Top view of the reader (stator), (**b**) bottom view of the reader, (**c**) photograph of the encoder, (**d**) envelope function providing the velocity, and (**e**) envelope function providing the motion direction. The nominal rotation speed is 60 rpm. Details of the materials used and dimensions are given in [20]. Reprinted with permission from [20]. Copyright 2018 IEEE.

The first electromagnetic encoder systems based on the amplitude modulation of a feeding harmonic signal caused by encoder motion were devoted to the measurement of angular displacements and velocities [15,18,19,20]. Such rotary encoders were first presented in [15], as an evolution of previously reported angular displacement and velocity sensors based on circular resonators (rotor) axially rotating over the stator, a circularly shaped transmission line [35,36,37]. In these angular displacement and velocity sensors based on the so-called axial configuration, the rotating resonator (typically an electric-LC resonator or an S-shaped split ring resonator) modulates the transmission coefficient of the line, as far as the electromagnetic coupling between the resonator and the line depends on their relative orientation [38]. This generates an AM modulated signal at the output port of the line, provided it is fed by means of a harmonic signal tuned to the resonance frequency of the resonant element. However, only two pulses per cycle are achieved by means of these axial angular displacement/velocity sensors. The reason is that angles differing in 180^o^ are undistinguishable in terms of the reflection and transmission coefficient, due to symmetry considerations [35,36,37]. Determining the rotation velocity from the time lapse between two adjacent pulses is possible, but these sensors are not able to provide the instantaneous velocity, in case it experiences changes within a cycle. For this reason, rotary sensors based on coupling modulation but providing a substantially higher number of pulses per revolution (PPR) were investigated [15]. The solution, as reported in [15], was simply to etch a high number of resonant elements along the perimeter of the rotor, a disc made of a dielectric material, forming a circular chain. As it was demonstrated in [15,18,19,20], the number of pulses per revolution can be twice the number of resonant elements of the circular chain, or chains. In [20], a double-chain electromagnetic rotary encoder with a total of 600 resonant elements (distributed in two chains) and able to provide 1200 pulses per cycle was reported. This incremental type rotary encoder was equipped with an additional non-periodic circular chain in order to determine the motion direction, either clockwise or counterclockwise, as given by the increasing or decreasing time distance between adjacent peaks of the non-periodic chain. The inclusions in [20] were rectangular-shaped metallic split ring resonators, able to modulate significantly the transmission coefficient of the transmission line of the stator, a coplanar waveguide (CPW) in [20], and consequently providing a relatively high modulation index in the AM modulated output signal. Without the purpose of providing details (which can be found in [20]), Figure 3 shows the photograph of the incremental rotary electromagnetic encoder with motion direction detection capability, based on a pair of velocity chains, and able to provide PPR = 1200, a competitive value. The figure includes also the response of the device as the rotor rotates at the velocity given in the caption.

The angular resolution in the rotary encoder of Figure 3 is 0.3°, as inferred from the number of pulses per revolution (PPR = 1200). Taking into account the diameter of the rotor (203.2 mm), the corresponding linear resolution is 0.53 mm. These values are competitive, but further decreasing the period of the chain, or chains, is very convenient to further improving encoder resolution. Linear encoders based on linear strips transversally oriented with regard to the axis of the encoder chain, and the corresponding reader, have been reported [23]. These systems provide an unprecedented resolution, as far as the period of the chains is as small as 0.6 mm. Incremental and absolute encoders based on such metallic linear inclusions have been reported, and will be the subject of the first subsection.

Metallic inclusions (either resonant elements or strips) can be subjected to mechanical friction and wearing, due to potential vibrations between the encoder and the sensitive element of the reader (stator). All-dielectric electromagnetic encoders, where the inclusions are made of a dielectric material with a dielectric constant substantially different to the one of the host substrate, constitute a good solution when mechanical wearing may jeopardize the functionality of the encoder [22]. An example of such permittivity contrast encoders will be also reported in this section.

Finally, another set of linear encoders included in this section concerns those solutions based on inclusions made of metallic patches [28]. In such encoders, the metallic patches alter the resonance frequency of the resonant element loading the transmission line of the reader. However, this frequency, and consequently the transmission coefficient at the frequency of the feeding signal/s, is perturbed regardless of the presence of cracks in the patches or mechanical wearing. Thus, these electromagnetic encoders are of interest as an alternative to all-dielectric encoders in applications where robustness against mechanical wearing is a key aspect.

### 3.1. Electromagnetic Encoders Based on Linear Strips

**Figure 4:**
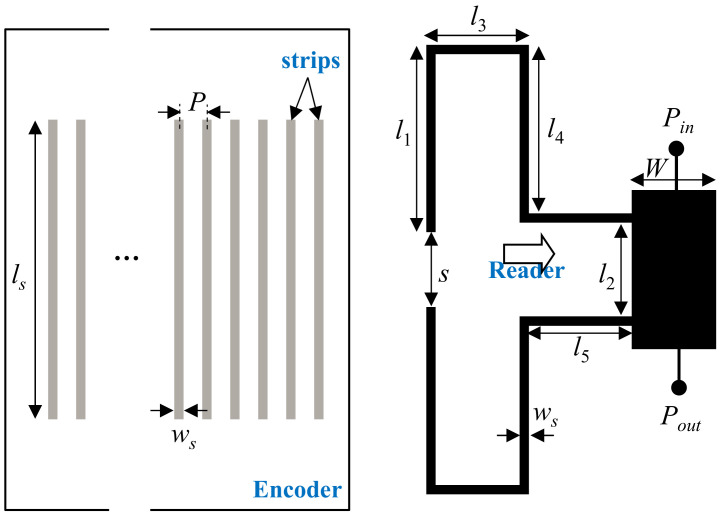
Topology of the encoder based on transversally oriented linear metallic strips, and topology of the reader, based on a pair of meandered stubs oriented face-to-face by the open ends (the arrow indicates the direction of motion). Reprinted with permission from [23]. Copyright 2019 IEEE.

In [23], incremental type electromagnetic encoders implemented by means of linear strips transversally oriented with regard to the axis of the encoder chain were proposed. The achieved resolution was 0.6 mm, i.e., the period of the (single) chain. Detection of such linear strips was achieved in [23] by means of a double stub loading a transmission line, as depicted in Figure 4. In such structure, the coupling between the meandered stubs can be neglected, as long as a strip of the chain does not lie on top of the open end of the stubs. However, covering the open end of the stubs by means of a strip activates inter-stub coupling, and the resonance frequency (visualized as a notch in the frequency response) splits into two frequencies. Moreover, the structure can be designed so that the first splitted frequency (notch) coincides with the pole of the bare (i.e., uncovered) double stub (see [23] for further details on the design). By this means, the excursion experienced by the transmission coefficient at that frequency is very large, resulting in a significant AM modulation factor.

**Figure 5:**
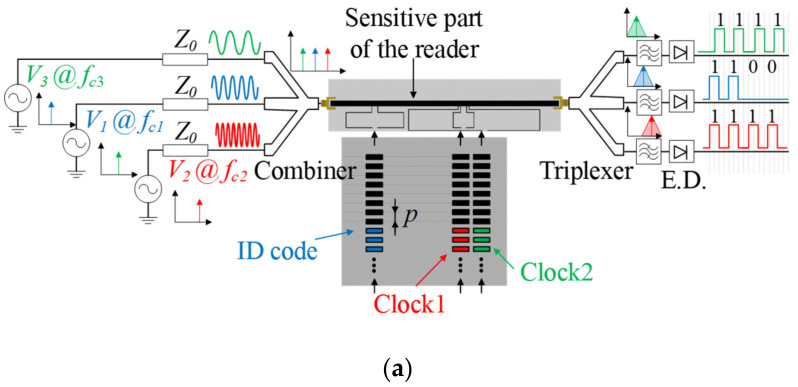

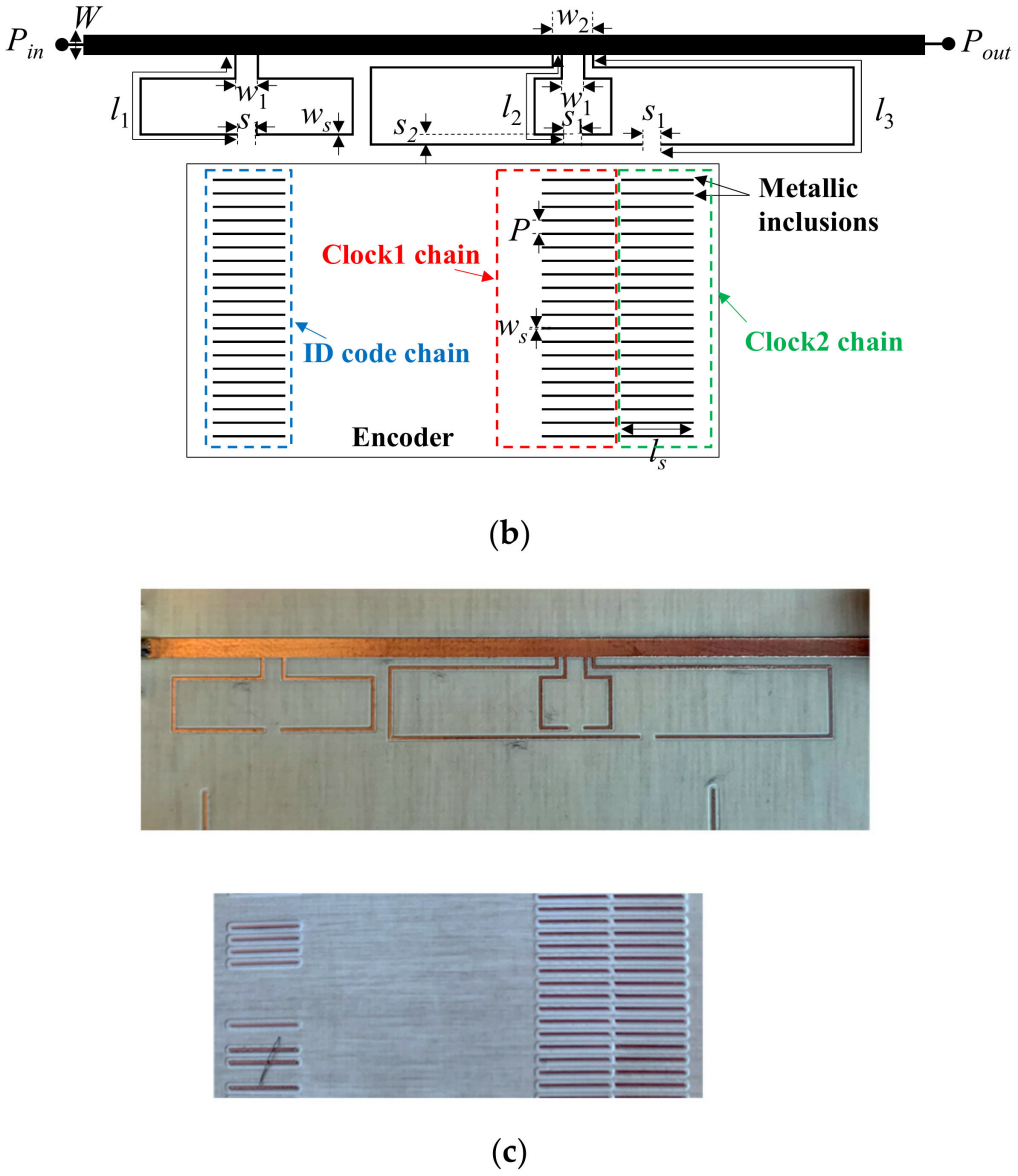
(**a**) Sketch of the absolute electromagnetic encoder system based on linear strips with motion direction detection capability; (**b**) Topology of the reader and encoder; (**c**) photograph of the Figure 1. 81, *l*_1_ = 24.39, *l*_2_ = 12.33, *l*_3_ = 48.60, *w*_1_ = 1.80, *w*_2_ = 3.40, *s*_1_ = 1.60, *s*_2_ = 0.90, *P* = 1.20, *w*_s_ = 0.20 and *l*_s_ = 6.40. The considered substrate for the reader is the *Rogers RO4003C* with dielectric constant *ε_r_* = 3.55, thickness *h* = 0.81 mm, and loss tangent tan*δ* = 0.0021. For the encoder, the only difference is the thickness, *h* = 0.204 mm. Reprinted with permission from [32]. Copyright 2021 IEEE.

In [23], the proposed encoders were implemented by means of a single chain of linear strips. Therefore, when all the strips are present the encoder can provide the relative instantaneous velocity with regard to the reader, as well as the incremental position (nevertheless, encoders with various ID codes were also implemented in [23], in order to demonstrate the potential of this system as a chipless radiofrequency identification (chipless-RFID) system with unprecedented data storage capacity based on time domain and sequential bit reading [33]). In [32], an absolute encoder system implemented by adding further double stubs to the host line of the reader, with an encoder based on three chains of linear strips, was reported. Moreover, such encoder system is able to provide the motion direction (that justifies the presence of the third encoder chain). The topology and photograph of the encoder and reader, as well as the sketch of the complete system, are depicted in Figure 5.

As it can be seen from Figure 5, the period of both clock chains is identical. Nevertheless, since such period does not coincide with the distance *s*_2_ (see Figure 5), one clock signal is either lagged or leaded with regard to the other, and therefore the direction of motion of the encoder can be detected. The three harmonic interrogation signals were tuned to the first resonance frequencies of the three double stubs, i.e., *f_c1_* = 2.02 GHz, *f_c2_*= 3.98 GHz and *f_c3_*= 0.959 GHz. To determine the absolute position of the encoder (actually the quasi-absolute position), the position chain (or ID code chain) should be encoded with the De Bruijn sequence, as indicated before. Therefore, it is necessary to read a bit of the coded chain, at a determined position, plus the previous *N*-1 bits in order to obtain the absolute position of the encoder, as specified before. In a real scenario, the system must include a table with the position assigned to each *N*-bit sub-code of the Bruijn sequence, this being necessary for both motion directions. In the event of a system reset, it is necessary that the encoder moves *N* strip positions in order to read the necessary *N* bits of the sub-code. For this reason, the proposed system should be designated as quasi-absolute (rather than absolute) position electromagnetic encoder. To give an idea of the number of bits necessary in a real situation, let us consider an encoder length of *L* = 1 m, and a period of *p* = 1.20 mm. In this case, according to expression (1), *N* = 10 bits suffice to determine the position. This means that the absolute position can be determined after a motion of the encoder corresponding to 1.2 cm.

**Figure 6:**
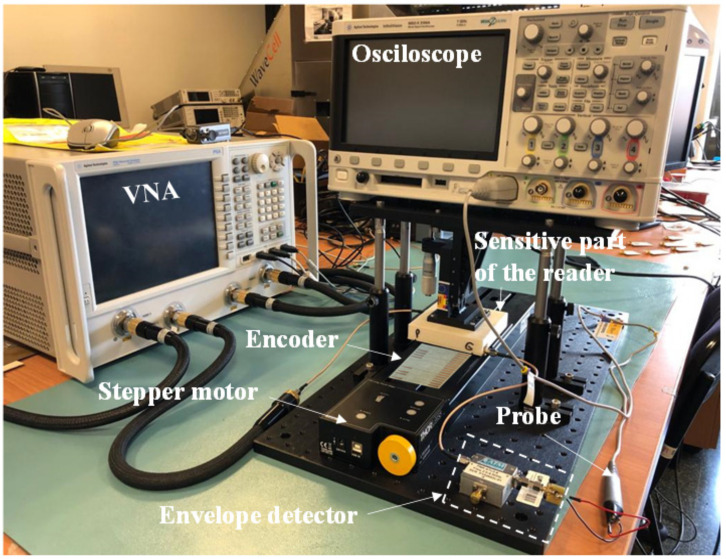
Photograph of the complete experimental setup providing the envelope functions.

**Figure 7:**
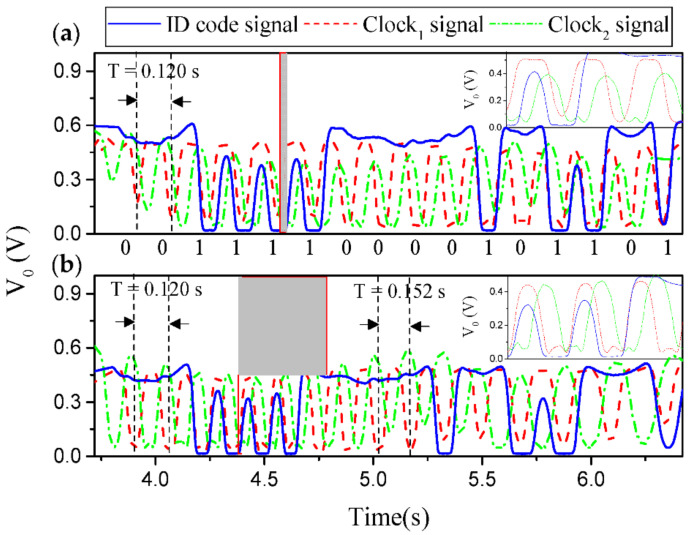
Measured envelope function of the 16-bit encoder by considering encoder motion with (**a**) constant velocity and (**b**) constant acceleration. The ID code is indicated. The estimated air gap in the measurements is 0.2 mm. Reprinted with permission from [32]. Copyright 2021 IEEE.

Nonetheless, a 16-bit encoder with *N* = 4 and a specific De Bruijn sequence was fabricated in [32] for validation purposes (Figure 5 depicts the photograph of such encoder). Figure 6 depicts the experimental setup for measurement purposes. The three measured envelope functions of the fabricated encoder, inferred by considering first constant velocity, and then constant acceleration of the encoder with regard to the sensitive part of the reader, are depicted in Figure 7a,b, respectively. The dips in the measured envelope function are indicative of the presence of metallic inclusions on top of the extremes of the stubs, at short distance. The reason is that when such strips are on top of the extremes of a double stub, the transmission coefficient is a minimum at the corresponding signal frequency. In Figure 7a the nominal velocity of the encoder is 10 mm/s. The redundant clock signal (Clock_2_) is advanced with regard to the clock signal (Clock_1_). From this information, the motion direction of the encoder can be inferred. The encoder velocity that results from the time lapse between adjacent pulses is found to be 10 mm/s, in excellent agreement with the nominal value. In Figure 7b, the envelope functions inferred by displacing the encoder over the sensitive part of the reader with a nominal constant acceleration of 3 mm/s^2^, are displayed. It can be seen that the encoder direction and the ID code (and thereby the absolute position) can be obtained regardless of the encoder velocity with regard to the sensitive part of the reader. These results validate the functionality of the system as a quasi-absolute electromagnetic position encoder, able to provide the instantaneous velocity (and acceleration), as well as the motion direction. The main relevant advantage of these encoders is spatial resolution (*p* = 1.20 mm), given by the chain period. The main drawback is related to the potential effects of strip wearing or friction, since encoder functionality is based on the integrity of the strips (i.e., damaged or fragmented strips may not be able to aid inter-stub coupling).

Other incremental encoders based on linear strips have been reported [21,39]. In [21], the reader is a gap coupled half-wavelength resonator. The presence of a strip on top of the half-wavelength resonator modifies the phase condition, thereby modifying the resonance frequency, and, consequently, the transmission coefficient at the frequency of the harmonic feeding signal. Nevertheless, the achieved modulation index is small, as compared to the one of the encoder system of Figure 5. The period of the encoder is 0.6 mm (identical to the one of the prototype in [23], and half the one of the system of Figure 5). However, the system is less tolerant to the effects of gap variations and misalignments between the encoder and the sensitive part of the reader. In the prototype presented in [39], the reader is a quarter-wavelength open-ended resonant stub, and the working principle is essentially the same as the one of the half-wavelength resonator. In this prototype, the encoders exhibit also a period of 0.6 mm, but robustness is not comparable to the one of the encoder system of Figure 5.

### 3.2. Electromagnetic Encoders Based on Dielectric Inclusions

In this section, an alternative to electromagnetic linear encoders based on metallic inclusions is discussed. As it was pointed out in the previous sub-section, metallic inclusions may suffer from damage (typically fragmentation or cracks), caused by wearing or unforeseen friction, that may limit their functionality. Essentially, the metallic inclusions modify the transmission coefficient of the reader line at the frequency of the harmonic feeding signal (signals in case of absolute encoders), with the effect of AM modulating the corresponding signal at the output port. However, this modulation of the transmission coefficient can also be achieved by means of dielectric inclusions, provided their dielectric constant is substantially different to the one of the host substrate of the encoder. With these all-dielectric permittivity contrast encoders, as they have been designated [22], the reader should be implemented by means of highly sensitive permittivity sensors, able to contactless detect the presence of dielectric inclusions on top of the sensitive region. Most highly sensitive microwave permittivity sensors are based on transmission lines loaded with resonant elements [40,41,42,43,44,45,46]. Thus, canonically, the readers for permittivity contrast electromagnetic encoders are implemented by means of transmission lines loaded with resonant elements [22]. Defect ground structure resonators, such as the complementary split ring resonator (CSRR) [47], or the slot resonator, among others, are good candidates as sensitive elements. Let us next review two different realizations of these type of encoders.

**Figure 8:**
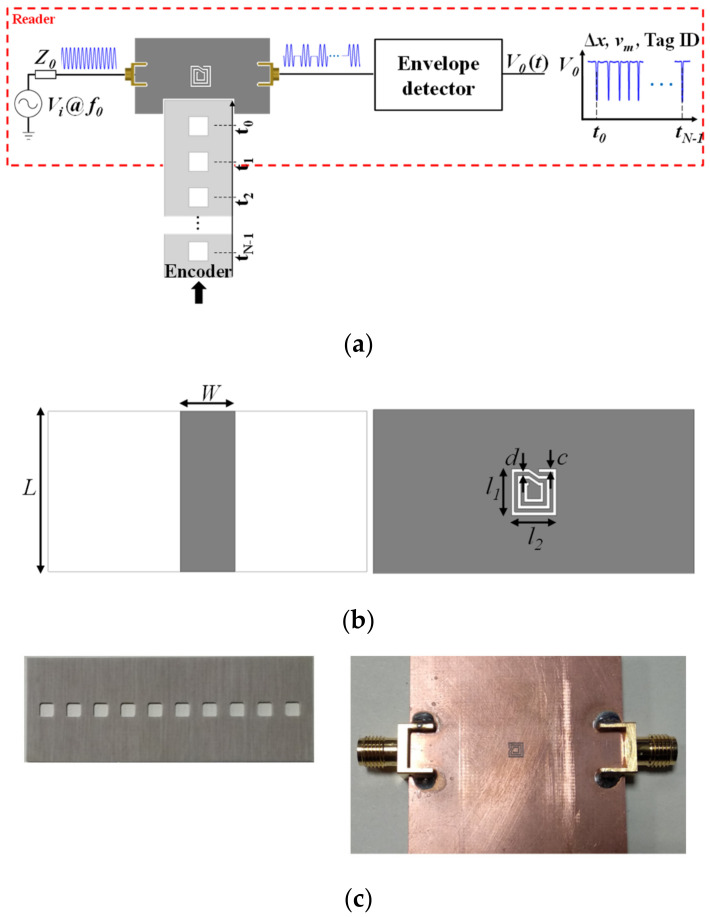
Sketch of the electromagnetic encoder based on square apertures (**a**), top and bottom view of the reader (**b**), and photographs of the fabricated encoder and reader (**c**). Dimensions are (in mm): *W* = 3.43, *L* = 10, *l_1_* = *l_2_* = 2.80, *c* = *d* = 0.20. The *Rogers RO4003C* with thickness *h* = 1.52 mm, dielectric constant *ε_r_* = 3.55, and loss tangent tan*δ* = 0.0027, is the considered substrate of the reader. Reprinted with permission from [22]. Copyright 2019 IEEE.

The first implementation was the first reported all-dielectric permittivity contrast encoder [22]. The dielectric inclusions are square-shaped apertures forming a linear chain on a host dielectric substrate of thickness *h* = 0.81 mm and dielectric constant *ε_r_* = 3.55. The encoder, depicted in Figure 8, is incremental, since it is based on a single chain of apertures. A microstrip transmission line loaded with a complementary spiral resonator (CSR) etched in the ground plane, beneath the conductor strip, constitutes the main part of the reader. The CSR was used in [22] because this resonant particle exhibits a relatively high quality factor (or, equivalently, a narrow notch bandwidth) [48]. A significant excursion of the transmission coefficient at the operating frequency is necessary to obtain a high modulation index, and this is achieved by means of a narrow notch. By achieving a high modulation index, the system exhibits robustness against potential vibrations or misalignments between the encoder and the reader, as discussed before. Another important aspect concerns the fact that CSRs are electrically small resonators [48]. This favors size reduction of the encoder, since the size of the inclusions (apertures) and the minimum period of the encoder are determined by resonator’s dimensions.

**Figure 9:**
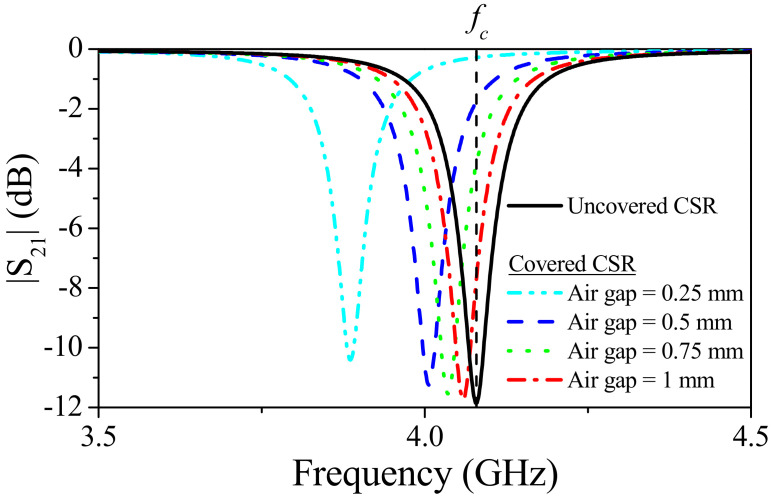
Simulated (by means of the *Ansys HFSS* commercial software) transmission coefficient of the bare complementary spiral resonator (CSR), and covered CSR by considering different air gaps. Reprinted with permission from [22]. Copyright 2019 IEEE.

The CSR-loaded microstrip line was designed with a notch frequency (uncovered CSR) at *f_c_* = 4.07 GHz, and with a characteristic impedance of the host line set to 50 Ω. The frequency response of the structure for the uncovered CSR and for the CSR covered with the material substrate of the encoder, located at various distances (air gap), are depicted in Figure 9. The narrow notch located at the above-cited frequency, *f_c_*, for the uncovered resonator shifts to the left when the CSR is covered. The notch position of the covered CSR depends, obviously, on the air gap distance. Thus, it is convenient to set the operating frequency of the system to the notch frequency for the uncovered CSR, *f_c_*. Therefore, the presence of apertures on top of the CSR in a reading operation should be revealed as dips in the envelope function.

**Figure 10:**
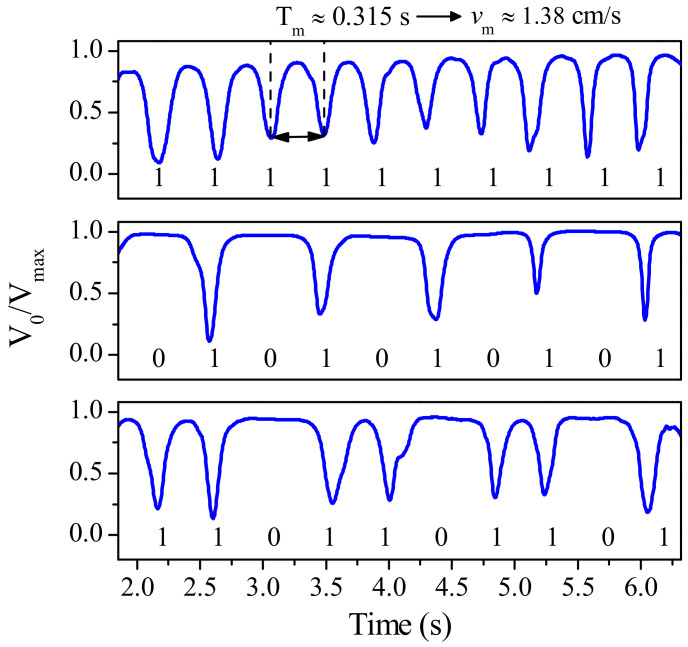
Measured envelope functions for the permittivity contrast electromagnetic encoder of Figure 8. The envelope functions with all the apertures present in the encoder (all bits set to “1”), as well as those envelope functions corresponding to the encoders with the indicated ID codes, are shown. Reprinted with permission from [22]. Copyright 2019 IEEE.

Figure 10 demonstrates the functionality of the encoder. Specifically, the envelope function of the encoder shown in Figure 8c (with all the apertures present at the corresponding positions, equivalent to all bits set to the logic state “1”), as well as those corresponding to other fabricated encoders (with the indicated codes), are depicted in the figure. Figure 10 provides the necessary information to extract the relative velocity and displacement between the encoder and reader. The distance between adjacent dips gives a velocity of roughly 1.38 cm/s. Let us mention that the carrier signal frequency was actually set to 4.02 GHz (rather than 4.07 GHz) in [22]. This is because the resonance frequency measured in the fabricated uncovered CSR was slightly shifted down, mainly due to fabrication related tolerances. However such displacement was less than 1.5%. Even though in the results of Figure 10, the envelope function for encoders with specific code are included, this encoder cannot be considered to be quasi-absolute, since the clock signal is not present. Nevertheless, in a hypothetical system with well-known encoder velocity, the determination of the absolute position would be possible, provided the whole encoder is codified according to the De Bruijn sequence, as discussed in the previous subsection, and provided the instants of time for encoder reading are determined from the encoder velocity and encoder period.

**Figure 11:**
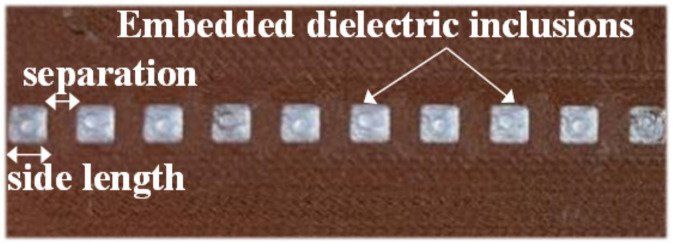
Permittivity contrast encoder based on embedded dielectric inclusions. The thickness of the encoders is 1 mm. Dimensions of the embedded square dielectric inclusions (in mm) are: 2.8 mm side length and 2.4 mm separation. Reprinted with permission from [30]. Copyright 2020 IEEE.

**Figure 12:**
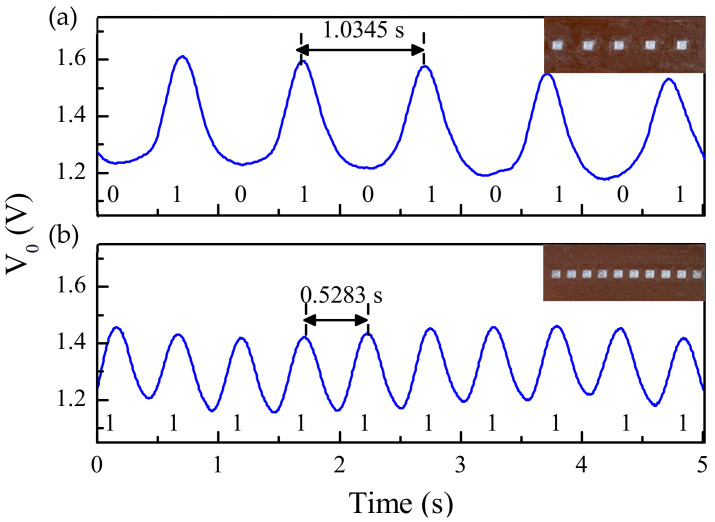
Measured envelope functions corresponding to the 10-bit microwave encoders implemented by means of embedded dielectric inclusions depicted in the inset, with ID codes “1010101010” (**a**) and “1111111111” (**b**). The harmonic feeding signal was tuned to *f_c_* = 3.9 GHz. Reprinted with permission from [30]. Copyright 2020 IEEE.

**Figure 13:**
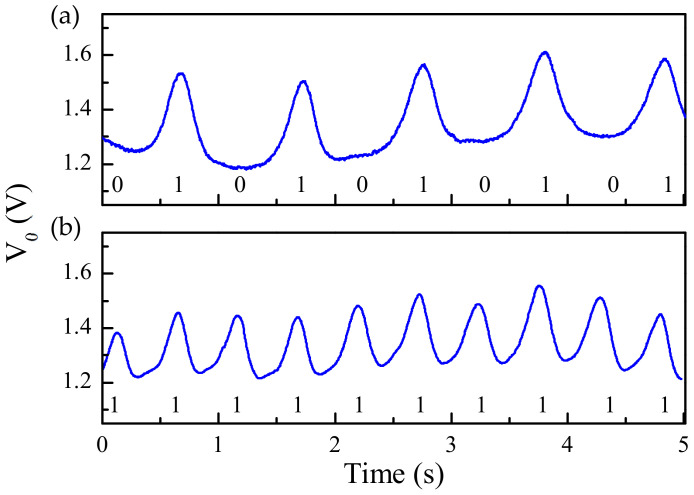
Measured envelope functions corresponding to the 10-bit encoders based on buried dielectric inclusions, with ID codes “1010101010” (**a**) and “1111111111” (**b**). Reprinted with permission from [30]. Copyright 2020 IEEE.

In [30], the apertures of the encoder of Figure 8 were replaced with dielectric inclusions. Such inclusions are made of a material with low dielectric constant as compared to the dielectric constant of the host substrate material. Two different filaments (PLA Polylactic acid and RS Pro MT-Copper) were used to implement the encoders by means of 3D printing. These materials exhibit a substantial contrast of their respective dielectric constants. The measured dielectric constant for PLA Polylactic acid was *ε_r_* = 3, whereas *ε_r_* = 7.6 was obtained for RS Pro MT-Copper. The dielectric permittivity contrast between both materials (defined as the ratio between the dielectric constants) is not as good as the one between vacuum (apertures) and the considered encoder substrate in Figure 8. Nevertheless, such combination of materials is adequate for the implementation of the permittivity contrast encoders, which can be read by means of a reader identical the one of Figure 8. The photograph of this encoder is depicted in Figure 11, whereas the measured envelope function is shown in Figure 12. The figure includes also the envelope function that results when the period is twice the period of Figure 11 (or, equivalently, the encoder is encoded with the ID code with sequence “101010…”). It was also demonstrated in [30] that it is possible to implement the same encoder with buried inclusions (and therefore invisible). The results, shown in Figure 13, are roughly undistinguishable from those of the encoder with visible embedded inclusions.

**Figure 14:**
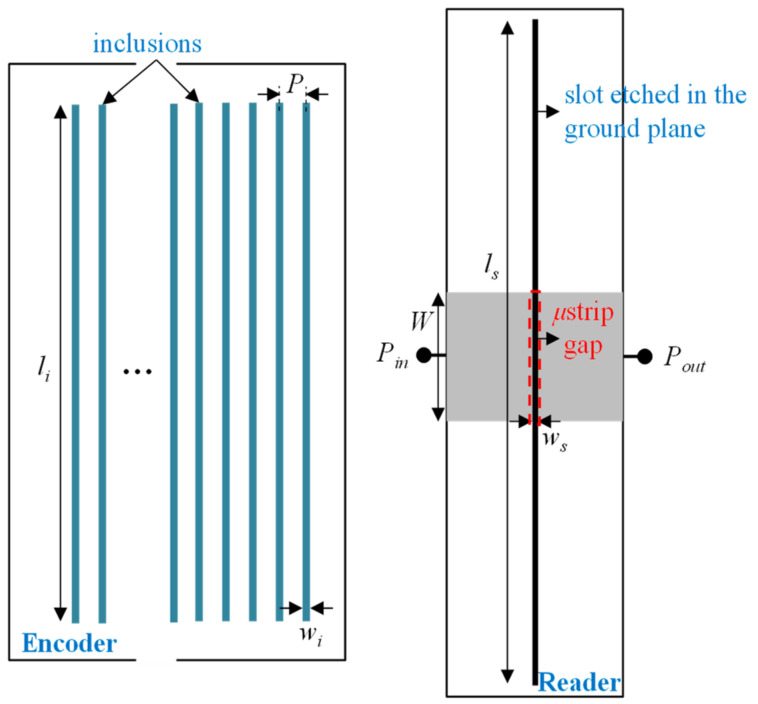
Topology of the reader-encoder, with encoder based on apertures or linear dielectric inclusions, and relevant dimensions (in mm). *W* = 3.98, *l_s_* = 24, *w_s_* = 0.2, *l_i_* = 18, *w_i_* = 0.4, and *P* = 3.4. Reprinted with permission from [25]. Copyright 2020 IEEE.

The main limitative aspect of the previous all-dielectric electromagnetic encoders is the relatively large period, providing a relatively poor resolution. In applications where high accuracy is required, the solution is the encoder system reported in [25], where the dielectric inclusions have a shape similar to the one of the linear strips of the encoders of the previous subsection. In [25], the reported encoders are incremental, whereas the absolute position encoder counterparts are reported in [27]. Let us review these encoders next. In the incremental encoder system, the encoder is made either of narrow apertures or embedded dielectric inclusions transversally oriented to the direction of the chain axis, see Figure 14. To properly detect such dielectric inclusions, a permittivity sensor consisting of a microstrip line with a transversely oriented slot resonator etched in the ground plane was proposed. Moreover, a gap is also etched in the line, at the same position of the slot resonator, since, by this means, the frequency response exhibits a pole to the left of the transmission zero caused by the slot resonator (see further details in [25]).

**Figure 15:**
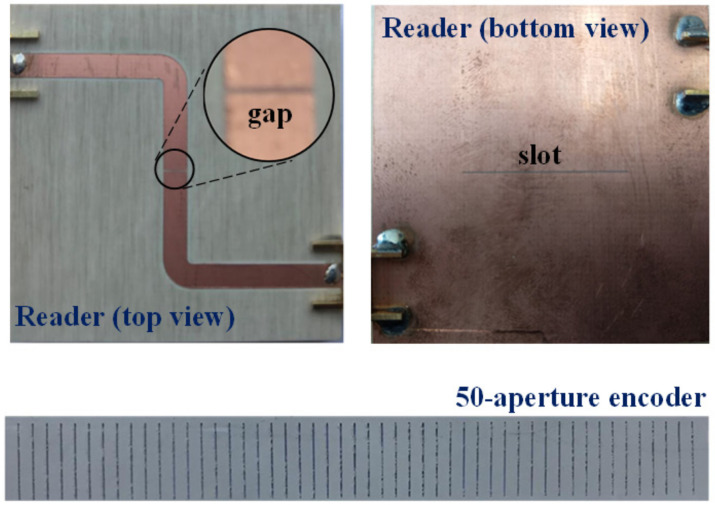
Photograph of the fabricated reader and encoder for the electromagnetic linear encoder system based on all-dielectric aperture encoders. Reprinted with permission from [25]. Copyright 2020 IEEE.

**Figure 16:**
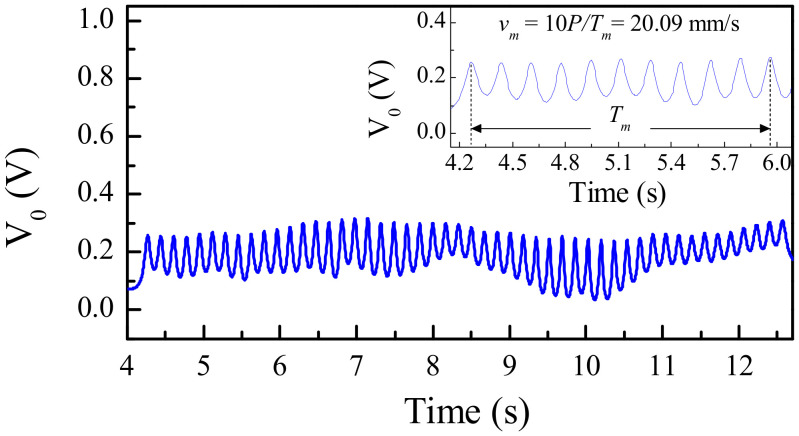
Measured envelope function for the fabricated 50-aperture encoder of Figure 15. Reprinted with permission from [25]. Copyright 2020 IEEE.

Figure 15 depicts the photograph of the fabricated reader, as well as the photograph of a fabricated 50-aperture encoder (implemented on the Rogers RO3010 substrate with thickness 0.635 mm, dielectric constant *ε_MUT_* = 10.2, and loss tangent tan*δ* = 0.0027). The substrate of the reader is the *Rogers RO4003* with thickness *h* = 1.524 mm, dielectric constant *ε_r_* = 3.55, and loss tangent tan*δ* = 0.0021. For encoder reading, the frequency of the interrogation signal was set to *f_c_* = 3.85 GHz, since at this frequency it was found that the excursion experienced by the transmission coefficient when the slot resonator is loaded or unloaded is maximum. The measured envelope function is depicted in Figure 16. Such envelope function provides the relative velocity between the encoder and the reader, since the encoder period is well known. The averaged velocity over 10 periods was found to be 20.09 mm/s, according to the results of Figure 16, i.e., very similar to the nominal value (20 mm/s).

**Figure 17:**
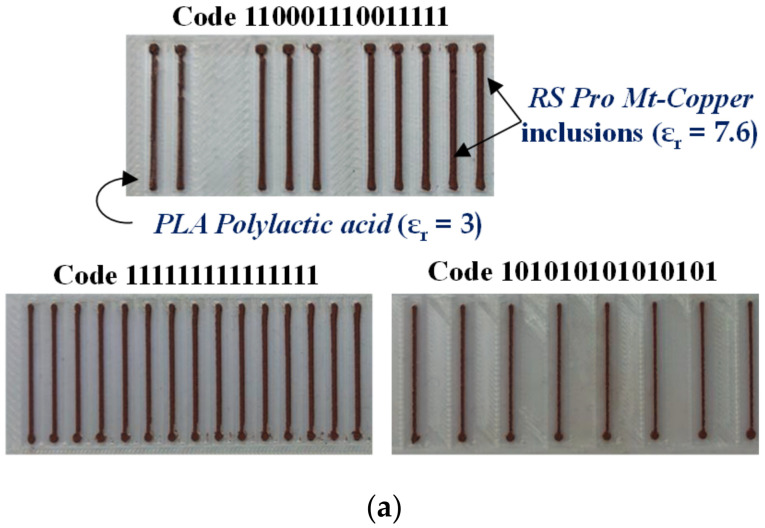

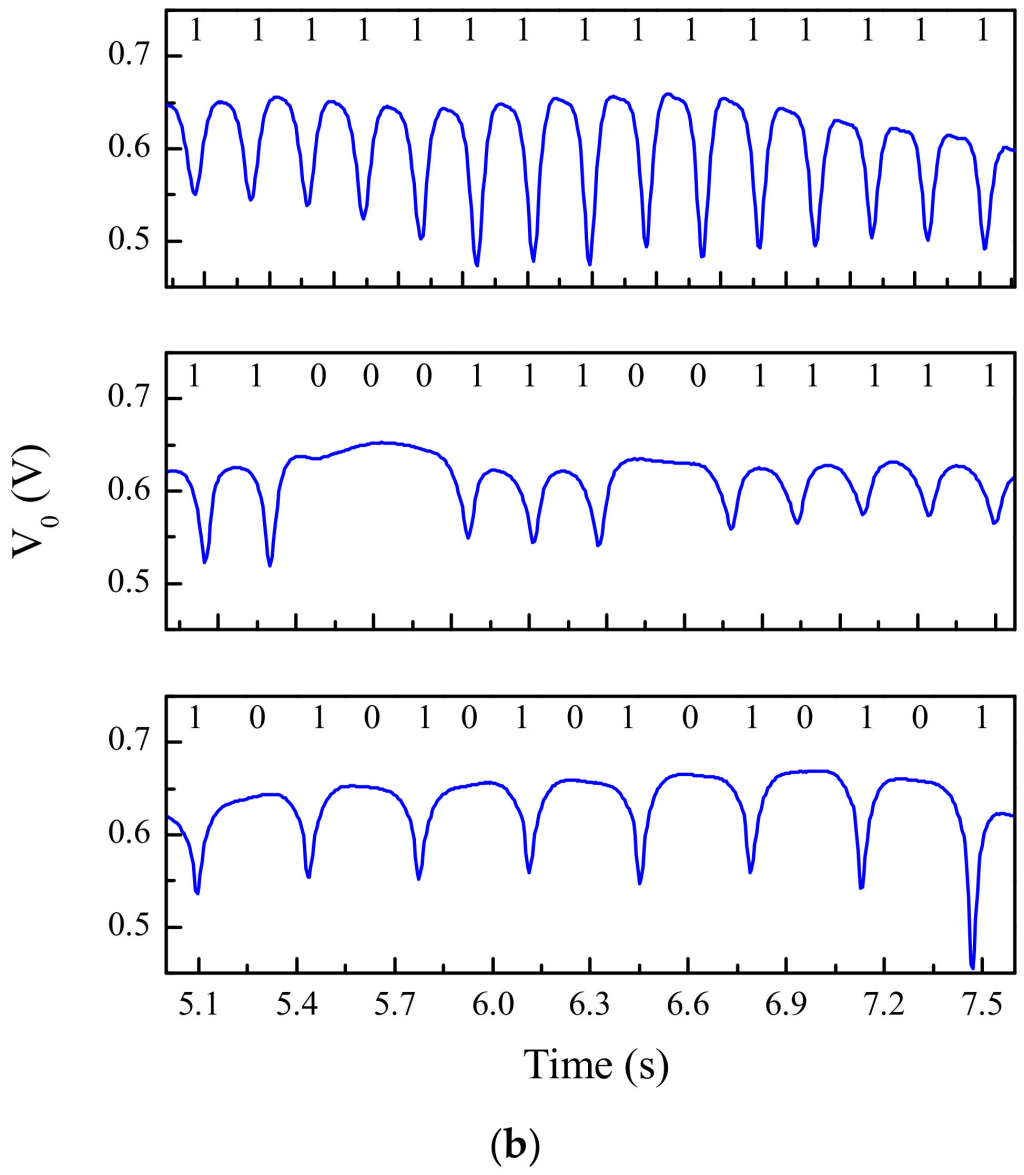
(**a**) Photograph of 15-bit encoders with the indicated ID codes; (**b**) corresponding envelope functions. The inclusions have a length of 18 mm and a width of 0.4 mm, whereas their separation is 3 mm (providing an encoder period of 3.4 mm). The estimated thickness of the 3D-printed substrate is 0.2 mm, and the estimated thickness of the dielectric inclusions is 0.2 mm. Reprinted with permission from [25]. Copyright 2020 IEEE.

In [25], all-dielectric permittivity contrast encoders fabricated by 3D printing were also characterized. As an example, Figure 17 depicts three encoders and the corresponding envelope functions. In these encoders, the inclusions are made of a high dielectric constant material as compared to the one of the host substrate. That is, the substrate was printed by using PLA Polylactic acid (with dielectric constant ε_r_ = 3), whereas the inclusions are strips of printed RS Pro MT-Copper (with dielectric constant *ε_r_* = 7.6). According to the results, the different encoders are correctly read. Nevertheless, in these encoders, the permittivity contrast is complementary to the one of the previous encoders. That is, the inclusions are made of a high dielectric constant material, whereas the dielectric constant of the substrate is comparatively small. Contrarily, in the previous encoders, the inclusions are apertures (with *ε_r_* = 1) made on a high dielectric constant material (substrate). This explains that the presence of inclusions on top of the slot resonator of the reader provides a dip, rather than a peak, in the envelope functions (see Figure 17), contrary to the encoders based on apertures.

**Figure 18:**
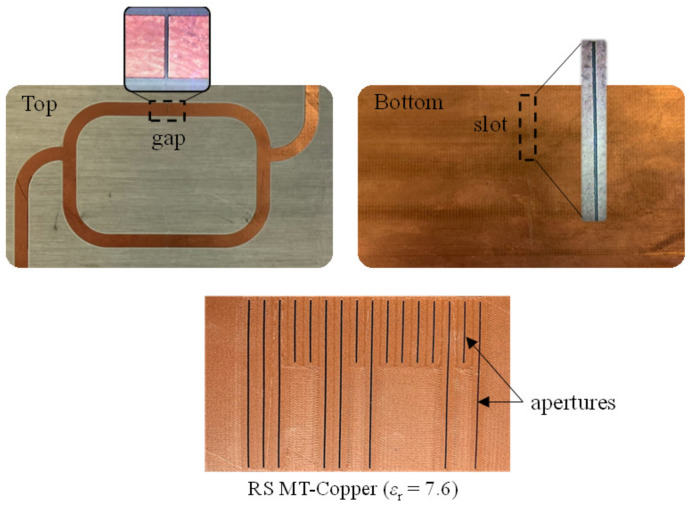
3D-printed quasi-absolute all-dielectric permittivity contrast position encoder and reader based on a pair of slot resonators [27].

**Figure 19:**
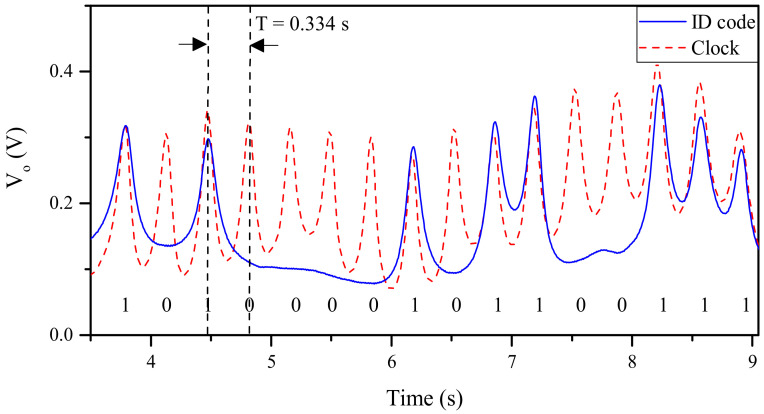
Measured envelope functions for the clock and ID code signals tuned to *f*_0*l*_ = 3.155 GHz and *f*_0*u*_ = 4.229 GHz [27].

The incremental aperture-based encoder of Figure 15 was equipped with an additional coded chain in order to synchronously read the ID code, and thereby obtain the absolute position (the ID code must follow the De Bruijn sequence as justified previously). However, for that purpose, the reader must be modified, and equipped with, at least, two resonant elements (slot resonators). This new reader and absolute encoder based on apertures are depicted in Figure 18 [27]. Actually, the encoder was 3D-printed using the RS Pro MT-copper as filament, whereas the reader was implemented on the substrate RO4003C (with dimensions, dielectric constant and loss tangent mentioned before). The measured clock signal and ID code of the 16-bit encoder of Figure 18 are depicted in Figure 19 (envelope functions). Such envelope functions were obtained by displacing the encoder over the sensitive part of the reader at 10 mm/s. The clock signal perfectly determines the time intervals for synchronous reading the De Bruijn sequence in the encoder chain (revealed as peaks in the envelope function). Hence, by this means, the absolute position of the encoder can be determined. Moreover, the measured relative velocity between the encoder and the sensitive part of the reader was found to be 10.17 mm/s (from the time interval between adjacent peaks of the clock signal). This value is in good agreement with the nominal value (10 mm/s). Note, however, that the displacement direction cannot be detected with this approach. The resolution of the encoder is good, but not as good as the one of the quasi-absolute encoder of Figure 5, made of metallic inclusions.

### 3.3. Electromagnetic Encoders Based on Metallic Patches

In this subsection, quasi-absolute electromagnetic encoders based on metallic patches are reviewed. The main difference as compared to the encoders reviewed in Section 3.1, based on metallic strips, is that the integrity of the patches is not critical, as it will be shown. By contrast, in the encoders of Figure 5, if there is a cut in the strips, the corresponding pulse in the envelope function does not appear. Thus, encoders based on metallic patches are of special interest in applications where wearing or mechanical friction cannot be avoided. Additionally, the functionality of encoders based on metallic patches is preserved by implementing the encoders on plastic or organic substrates (e.g., paper), or, even, on rubber material. Moreover, it is also possible to use conductive inks in order to print the patches (e.g., by means of industrial processes, such as screen-printing or offset, or by means of inkjet printing) on the considered substrate material. This robustness and the possibility of considering various substrate types make these encoders based on metallic patches firm candidates in many industrial applications.

**Figure 20:**
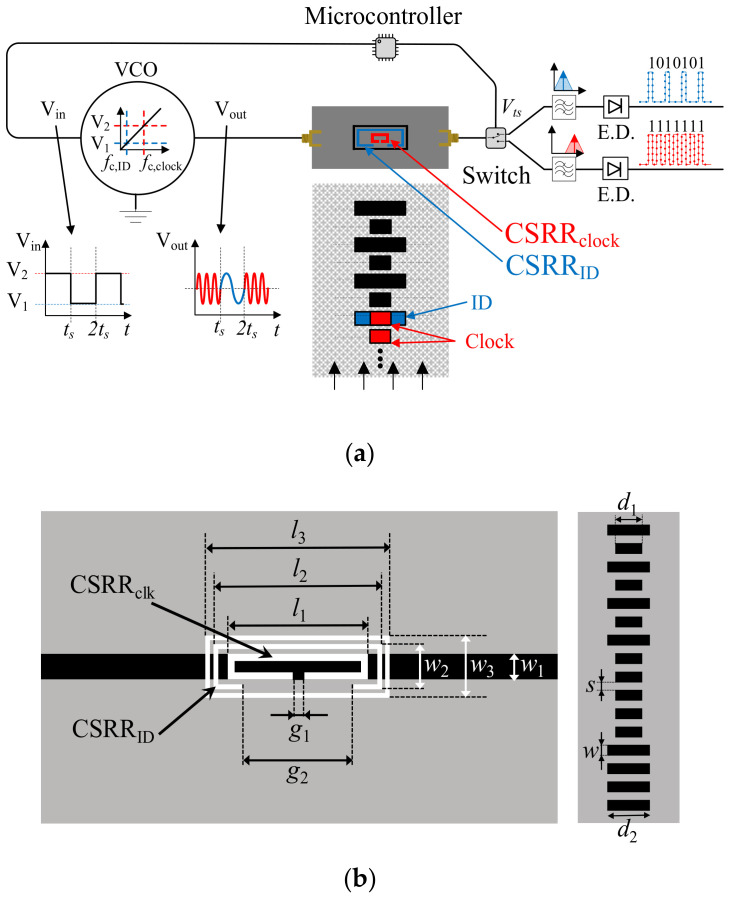
Sketch of the quasi-absolute encoder based on a single chain of metallic patches (**a**) and layout of the reader and encoder (including relevant dimensions) (**b**). Relevant reader dimensions are *l*_1_ = 10.3 mm; *l*_2_ = 12.3 mm, *l*_3_ = 13.5 mm, *w*_1_ = 1.9 mm, *w*_2_ = 3.1 mm, *w*_3_ = 4.3 mm, *g*_1_ = 1.0 mm, *g*_2_ = 7.2 mm. CSRR slots width are *c*_1_ = 0.5 mm and *c*_2_ = 0.3 mm for the inner and the outer CSRR, respectively, and the outer slot ring width is *c* = 0.3 mm. Encoder dimensions are *d*_1_ = 9.3 mm; *d*_2_ = 14.5 mm, *s* = 3 mm and *w* = 3 mm. Reprinted with permission from [26]. Copyright 2020 IEEE.

**Figure 21:**
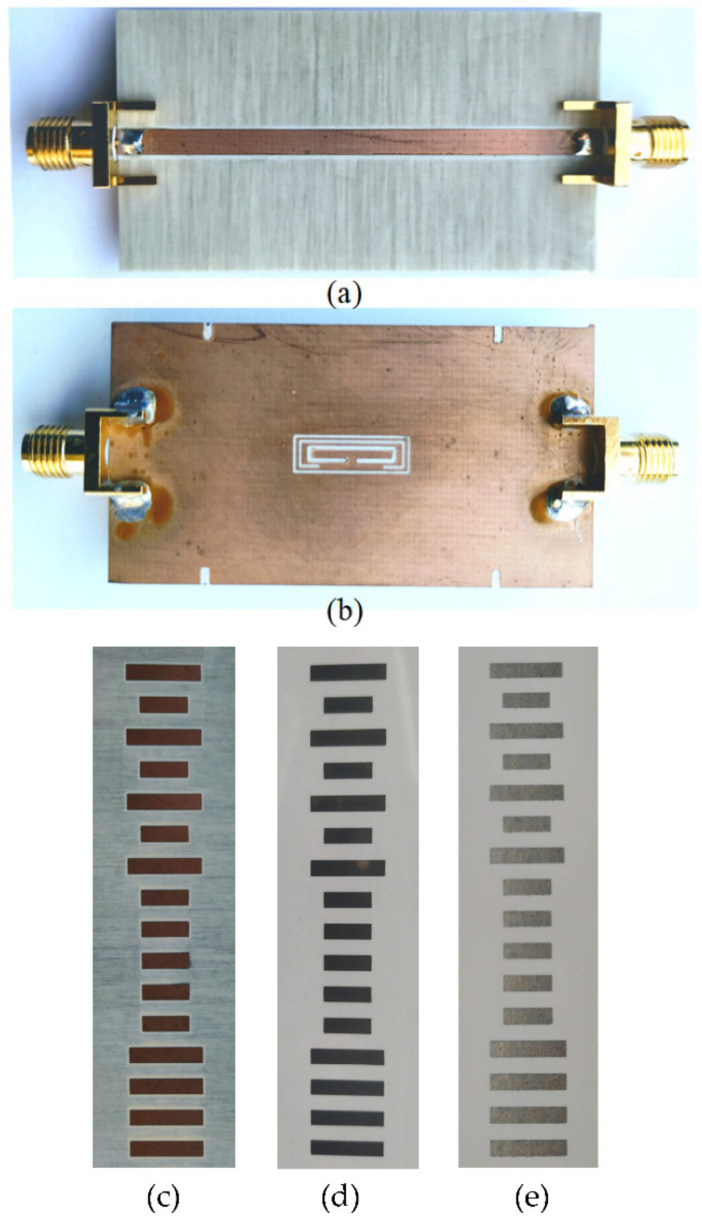
Photograph of the top (**a**) and bottom (**b**) view of the reader, implemented on the Rogers RO4003C with dielectric constant *ε_r_* = 3.38, thickness *h* = 0.81 mm and loss factor tanδ = 0.0022, and photograph of an encoder coded with the De Bruijn sequence and implemented in different substrates: (**c**) RO4003C with dielectric constant *ε_r_* = 3.38, thickness *h* = 0.2 mm and loss factor tanδ = 0.0022, (**d**) plastic substrate (polyethylene naphthalate, or also known as *PEN*, with a thickness *h* = 125 μm, from *Dupont*), and (**e**) paper substrate (powercoat *XD* with a thickness *h* = 200 μm, from Arjowinngs). Reprinted with permission from [26]. Copyright 2020 IEEE.

Two main prototypes of quasi-absolute encoders are reviewed in this section. In one case, synchronously reading the ID code is achieved by means of a single coded chain [26]. This is an interesting solution since one encoder chain is avoided, but the direction of motion cannot be determined. In the second considered approach, two encoder chains are used [28]. One is devoted to the absolute position (the coded one), whereas the other one provides the clock, the encoder velocity and the motion direction. Concerning the first prototype, the chain is made of rectangular patches, and the code is given by the size of the patches. The reader is based on a microstrip line loaded with two different resonators (CSRRs), one inside the other. The idea behind this approach is very simple. The small resonant element is sensitive to all the patches, regardless of its size. Therefore, the harmonic signal tuned to the resonance frequency of this smaller resonator provides the clock signal. By contrast, the larger CSRR is only sensitive to the larger patches. Thus, the AM modulated signal with carrier frequency tuned to the resonance frequency of the larger resonator provides the ID code. The sketch of the working principle and layout of the reader and encoder are shown in Figure 20, whereas the photograph of the reader, and photograph of three identical encoders, but implemented on different substrates (commercial microwave substrate, plastic, and paper) are depicted in Figure 21.

**Figure 22:**
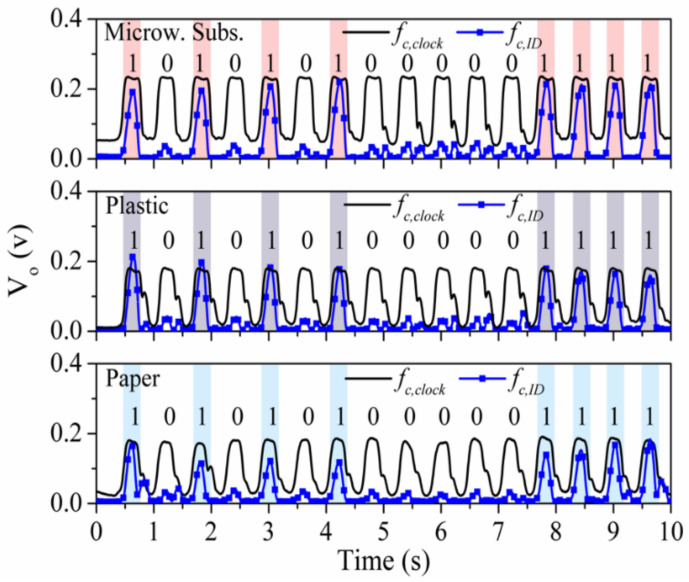
Envelope functions (clock and ID signals) inferred by reading the fabricated encoders in microwave substrate, in plastic substrate and in ordinary paper. Reprinted with permission from [26]. Copyright 2020 IEEE.

The frequencies of the interrogation harmonic signals were tuned to *f_c,clock_* = 5.31 GHz and *f_c,ID_* = 4.63 GHz. Figure 22 depicts the responses (envelope functions) of the different measured encoders of Figure 21, including the clock signals and the ID code signals. As expected, the peaks in the ID signals perfectly correlate with the logic state “1” of the corresponding ID code (corresponding to larger patches). Moreover, the number of peaks in the clock signal coincides with the number of patches. These results validate this quasi-absolute position encoder. It is worth mentioning that the system is functional even by considering the implementation of the encoders on plastic and paper substrates (by means of inkjet printing, using the Orgacon Nanosilver Inkjet Ink from the AGFA). Thus, according to these results, it can be concluded that neither the substrate material, nor the conductive material, are critical aspects in these quasi-absolute encoders based on metallic patches. Note that the conductivity of the inkjet-printed patches is of the order of ten times smaller than the one of copper, used in the encoder implemented on the commercial microwave substrate.

**Figure 23:**
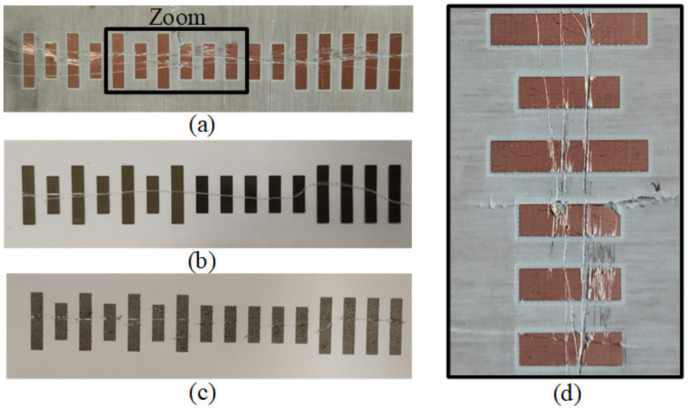
Photograph of the tags with deliberately generated cracks implemented on microwave substrate (**a**), plastic substrate (**b**), paper (**c**), and zoom view of cracked patches of the microwave substrate (**d**). Reprinted with permission from [26]. Copyright 2020 IEEE.

**Figure 24:**
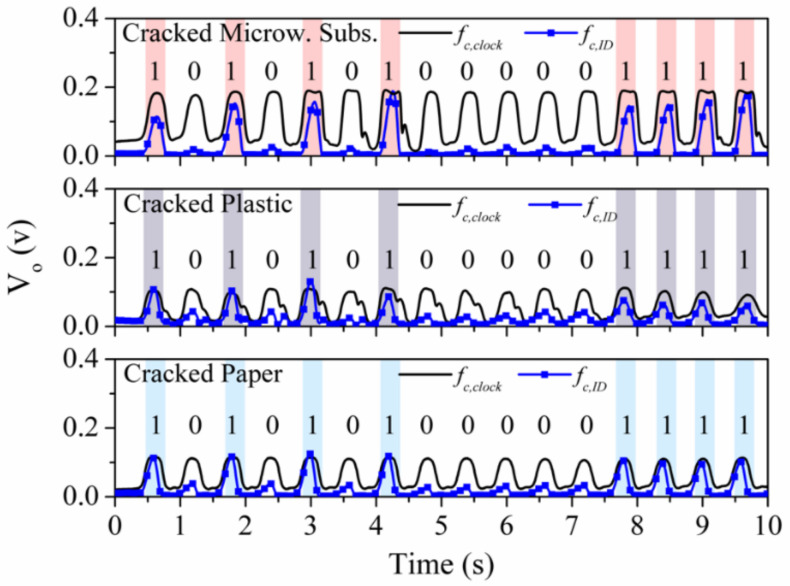
Clock and ID signals inferred by reading a microwave substrate, plastic, and paper-based encoder with cracks in all patches. Reprinted with permission from [26]. Copyright 2020 IEEE.

In [26], the robustness of the encoders based on metallic patches against wearing or friction was studied. Indeed, the resonance shift caused by a metallic patch is not affected if small (unexpected) cuts or cracks (that may appear as consequence of mechanical wearing) are present in the patch. Nevertheless, if excessively large cracks or a significant damage in the metal layer of the patch is generated (e.g., by extreme friction), loss of encoder functionality can be expected. To demonstrate this robustness of the proposed tags against wearing/friction, transverse cuts in the fabricated encoders were deliberately generated in [26], as depicted in Figure 23. The responses of these encoders with cracks are depicted in Figure 24. The obtained envelope functions reveal that the periodic peaks in the clock signals are perfectly visible, and the ID code signal for each cracked tag perfectly provides the ID code. Thus, despite the presence of cracks in the patches, system functionality is preserved.

**Figure 25:**
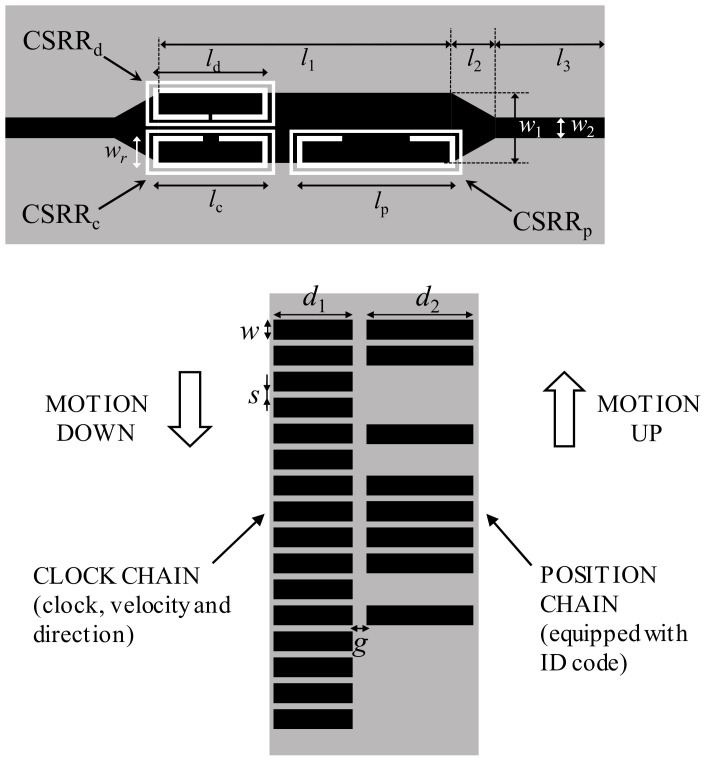
Layout of the reader and encoder of the absolute encoder system based on metallic patches, with motion direction detection capability. The total area of the encoder is 30 × 70 mm^2^, with the following metallic patch dimensions: *d*_1_ = 11.5 mm; *d*_2_ = 15.9 mm, *w* = 3 mm, *s* = 1 mm and *g* = 1.9 mm. Reader dimensions are: *l*_1_ = 26.6 mm; *l*_2_ = 3.8 mm, *l*_3_ = 10 mm, *w*_1_ = 6.4 mm, *w*_2_ = 1.9 mm, *l*_c_ = *l*_d_ = 10.5 mm; *l*_p_ = 14.5 mm, *w_r_* = 2.9 mm. CSRR slots width is *c* = 0.5 mm, and ring splits are *s*_d_ = 0.4 mm, *s*_c_ = 1.6 mm and *s*_p_ = 6.2 mm. The encoders were implemented in the Rogers RO4003C substrate with thickness *h* = 0.81 mm, dielectric constant *ε_r_* = 3.38, and loss factor tanδ = 0.0022. The reader substrate is identical but with a thickness of *h* = 0.2 mm. Reprinted with permission from [28]. Copyright 2020 IEEE.

The second prototype of quasi-absolute encoder based on metallic patches, reported in [28], is able to provide the motion direction. Two chains are present in the encoder, the position chain (codified with a certain ID code following a De Bruijn sequence), and the clock/velocity chain, the latter providing also the motion direction. Rectangular patches constitute both chains, and the ID code of the position chains is determined by the absence or presence of patch. The layout and photograph of the reader and encoder are depicted in Figure 25. Three resonant elements (CSRRs) are used as sensing elements in the reader. One of these CSRRs is devoted to the position chain (CSRR_p_), another one to the clock signal (CSRR_c_), and the third one (CSRR_d_) to the redundant clock signal necessary to determine the direction of motion (from the lag or led with regard to the other clocks signal).

**Figure 26:**
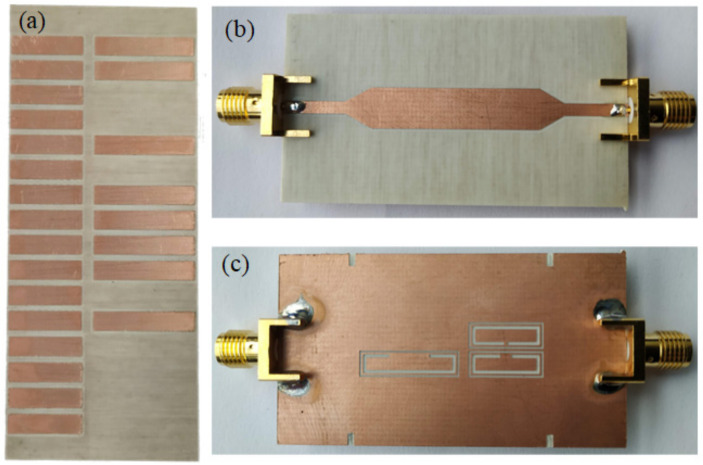
Photograph of the fabricated encoder (**a**) and top (**b**) and bottom (**c**) view of the reader. The ID code of the encoder is “1100-1011-1101-0000”. Reprinted with permission from [28]. Copyright 2020 IEEE.

**Figure 27:**
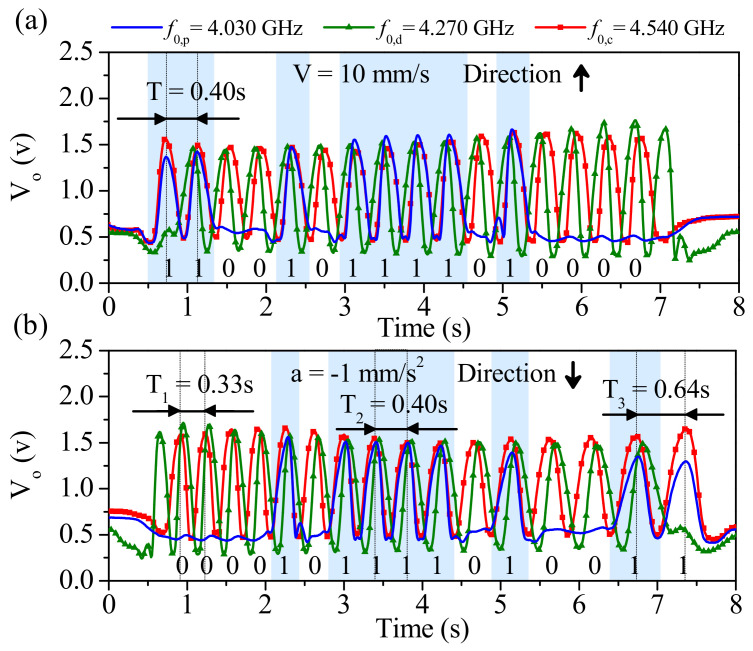
Measured envelope functions for (**a**) encoder motion up and *v* = 10 mm/s; (**b**) encoder motion down with constant acceleration of *a* = −1 mm/s^2^. The considered vertical distance, or air gap, between the encoder and the reader is 1 mm in both cases. Reprinted with permission from [28]. Copyright 2020 IEEE.

The photographs of the fabricated encoder and reader are shown in Figure 26, whereas Figure 27 depicts the three envelope functions that result from the three interrogation signals. The frequency of such signals was tuned to *f*_0,p_ = 4.030 GHz, *f*_0,d_ = 4.270 GHz, and *f*_0,c_ = 4.540 GHz, respectively, for the position, motion direction and clock signals. It follows from Figure 27 that the synchronism between the clock signal and the envelope function providing the ID code arise. The “1” logic states in the ID code are revealed as peaks in the envelope function. In Figure 27a the encoder is displaced upwards at a velocity of 10 mm/s (according to the scheme of Figure 25), so that the clock-chain patches first cross the CSRR_c_ and then the CSRR_d_. Consequently, the redundant envelope function should be delayed with regard to the clock signal. This is exactly what occurs, and therefore the direction of motion is correctly predicted). From the distance between adjacent pulses in the clock signal (*T* = 0.40 s), and taking into account the encoder period (*p* = 0.4 cm), the encoder velocity is found to be 10 mm/s, i.e., in good agreement to the nominal value (10 mm/s).

The envelope functions inferred by displacing downwards the encoder with a constant (negative) acceleration of −1 mm/s^2^ are depicted in Figure 27b. The clock-chain patches first cross the CSRR_d_ and then the CSRR_c_. The measured instantaneous velocities corresponding to time lapses between different pairs of adjacent pulses (i.e., *T_1_* = 0.33 s, *T_2_* = 0.40 s and *T_3_* = 0.64 s) were obtained. The corresponding velocities are *v_1_* = 12.12 mm/s, *v_2_* = 10 mm/s and *v_3_* = 6.25 mm/s. Therefore, the resulting acceleration −1.01 mm/s^2^, in good agreement to the nominal value. With these results, the functionality of the system is validated.

**Figure 28:**
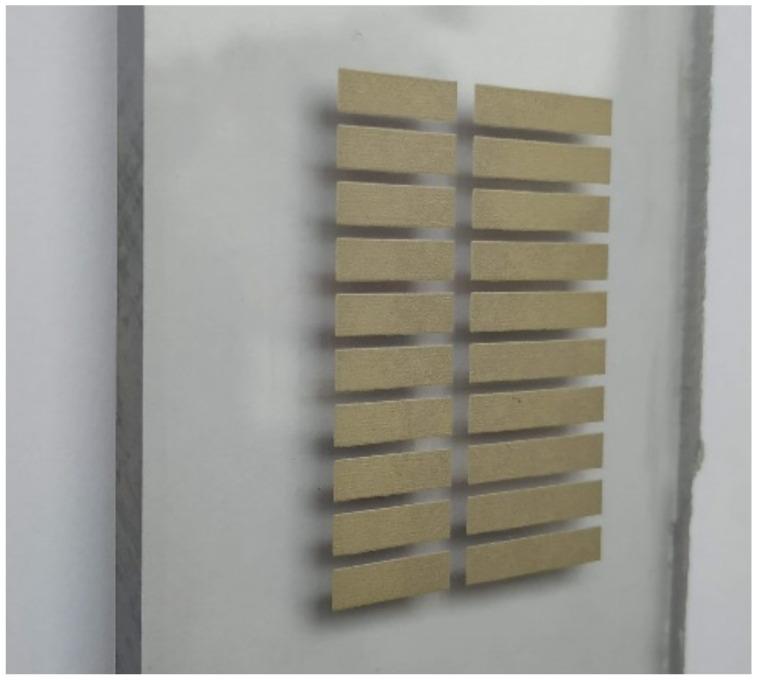
Photograph of a quasi-absolute 10-bit encoder implemented on polycarbonate. Patch dimensions are those indicated in Figure 25.

**Figure 29:**
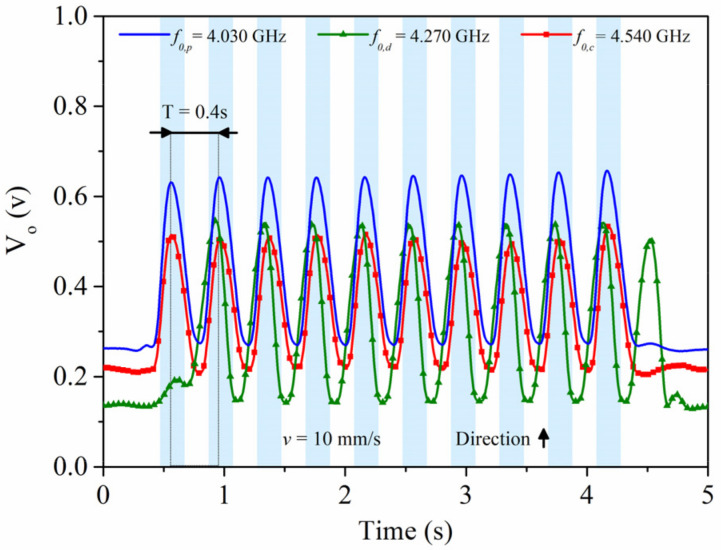
Measured envelope functions for encoder motion at a constant velocity of *v* = 10 mm/s in the upper direction (according to the sketch of Figure 25), corresponding to the encoder implemented on polycarbonate of Figure 28. The considered vertical distance between the encoder and the reader is 1 mm.

To demonstrate the versatility of the quasi-absolute encoder of Figure 26, a 10-bit encoder (with all bits of the ID code chain set to “1”) was screen-printed on a 5-mm thick polycarbonate substrate (Figure 28). For that purpose, the Narcote ELG commercial conductive ink was used. The measured envelope functions, depicted in Figure 29, reveal that the encoder is also functional when it is implemented on such substrate.

## 4. Comparative Analysis and Discussion

Let us briefly compare in this section the different discussed electromagnetic linear encoders. In terms of position resolution, the most competitive encoders are those based on linear strips. For example, the period of the quasi-absolute position encoders of Figure 5 is 1.2 mm, but the incremental encoder reported in [23] based on an identical (double stub) reader exhibits a period of 0.6mm. By contrast, the permittivity contrast encoders of Section 3.2, or the encoders based on metallic patches discussed in Section 3.3, do not exhibit such good resolution. For permittivity contrast encoders, the sensitive part of the reader is essentially a dielectric constant sensor (typically implemented by means of defect ground structure resonators, e.g., a slot resonator, a CSR, a CSRR, etc.). Such sensors are sensitive not only to the material on top of them, but also to the properties of the surrounding region (with an area of influence determined by the limits of the fringing fields of the resonant sensing elements). This explains that in order to detect the dielectric inclusions from the host medium, longer periods are needed. However, these all-dielectric permittivity contrast encoders (especially those based on apertures) are very robust against mechanical wearing or friction, as far as the encoders are not implemented by means of metallic inclusions. The functionality of the encoders based on metal patches is not based on the integrity of such patches, contrary to the encoders based on linear strips. The metal patches alter the resonance frequency of the sensing resonator, similar to dielectric inclusions. For this reason, the resolution of such encoders is worse than the one obtained in encoders implemented by means of linear strips. However, as it has been shown in Section 3.3, encoders based on metallic patches are robust against the presence of cracks (e.g., caused by mechanical friction). One interesting aspect of encoders based on metallic patches concerns the fact that such encoders can be implemented by means of additive processes, such as screen-printing or inkjet, on various type of dielectric substrates, including not only commercial microwave substrates, but also plastic substrates (e.g., PET, polycarbonate), paper, and even rubber. Thus, high versatility is achieved with this approach, of interest for industrial applications. For example, in applications such as conveyor belts, elevators, or any other industrial process where relatively large encoders are needed, implementation in low cost substrates is a due in order to reduce costs. Thus, solutions based on plastic substrates or rubber, with screen-printed encoders (either incremental-type or quasi-absolute), may be of interest. As an example, let us mention that the authors have demonstrated the functionality of quasi-absolute encoders (with 0.5 cm resolution and focused on large scale position measurements) implemented in rubber, a low-cost material of interest in many applications.

Table 1 summarizes a comparison of the different considered electromagnetic encoders, including other encoders not reported in this review paper. The main relevant (quantitative) parameter is encoder resolution. Nevertheless, other important aspects such as robustness against wearing and friction, encoder type (incremental or quasi-absolute), and the considered encoder substrate material, are included in the table.

It should be mentioned that the proposed encoders can also be of interest as chipless-RFID tags operating in time domain, where the bits are read sequentially by proximity (through near field) [33,49,50,51,52,53,54,55]. In this case, the resolution must be as small as possible, in order to accommodate the largest possible number of bits in the minimum space, and, for this main reason, the solutions based on linear metallic strips are the preferred ones. Such chipless-RFID systems, based on time-domain signature barcodes (the designation of the electromagnetic encoders as chipless-RFID tags), are of special interest in applications where reading by proximity does not represent a drawback, or, even, may be convenient in order to offer a high level of confidence against eavesdropping or spying. Secure paper and authentication of premium products are target applications of these chipless-RFID systems, where the main advantage over other chipless-RFID approaches (e.g., [56,57,58,59,60,61,62,63,64,65]) concerns the data storage capacity, only limited by tags size in electromagnetic encoders (for example, 100-bit tags with a data density of 26.04 bit/cm^2^ were reported in [23]).

Future investigations on electromagnetic encoders should include the development of a complete system incorporating the necessary electronics for harmonic generation and post-processing. Work is in progress to the development of a large scale position sensor based on quasi-absolute electromagnetic encoders screen-printed in rubber material.

To end this section, let us mention that the comparison in Table 1 is restricted to electromagnetic encoders. The reason is that comparing such encoders with other encoder types, such as optical [1,2,3,4,5,6,66,67] or magnetic encoders [7,8,9,10,11,12,13,14], including devices based on the giant magnetoimpedance effect [68,69,70], with completely different principles, is not representative. As mentioned in the introduction, the electromagnetic encoders subject of this review paper are not competitive against optical encoders in terms of resolution, but the robustness against the presence of dust, grease, etc., is superior in electromagnetic encoders. As compared to magnetic based encoders, the devices of this paper are of extreme simplicity and cost.

## 5. Conclusions

In summary, this review paper has been focused on the design and applications of electromagnetic encoders, of interest as position and velocity sensors. The working principle of such encoders has been presented, and then several types of encoders, classified according to the considered inclusions (metallic strips, dielectric inclusions, or metallic patches) have been reviewed and compared. Prototypes of incremental encoders, as well as quasi-absolute encoders, the latter being able to provide the absolute position and, in some cases, the motion direction, have been reported in the paper. The reported resolutions are not as good as those achievable with optical encoders. However, operating at microwaves, electromagnetic encoders are robust against harsh environments, e.g., subjected to pollution, dirtiness, or grease. As compared to magnetic encoder systems, typically based on magnets or inductive elements, the reviewed electromagnetic encoder systems are very simple, since the reader consists merely on a transmission line loaded with a planar resonator (or various resonators), the sensitive element, whereas the encoder is a chain (or chains) of metallic or dielectric inclusions in a host dielectric substrate. Encoder functionality in various types of substrates (including microwave substrates, plastic and paper) has been demonstrated, and it has been shown that for encoders based on metallic inclusions, particularly metallic patches, the functionality is preserved despite the fact that the patches are damaged (e.g., in the form of cracks eventually generated by mechanical wearing or friction). Thus, these electromagnetic encoders are highly versatile and can be of interest in multiple industrial applications, as an alternative to optical and magnetic encoders. The cost of electromagnetic encoders is also a competitive advantage, as far as the associated electronics in these encoders is simple, and these encoders are based on simple readers. Moreover, the movable part can be implemented in low-cost substrate materials.

## Figures and Tables

**Table 1:** Comparison of various electromagnetic encoders.

Encoder	Type	Resolution	Robustness ^1^	Material ^2^
Figure 3	Rotary/Incremental	0.3°	Poor	Cu/Microwave
Figure 5	Linear/Absolute	1.2 mm	Poor	Cu/Microwave
Figure 8	Linear/Incremental	5.2 mm	Good	Air/Microwave
Figure 11	Linear/Incremental	5.2 mm	Good	PLA/RS Pro MT-Copper
Figure 15	Linear/Incremental	3.4 mm	Good	Air/Microwave
Figure 17	Linear/Incremental	3.4 mm	Good	RS Pro MT-Copper/PLA
Figure 18	Linear/Absolute	3.4 mm	Good	Air/Microwave
Figure 21c	Linear/Absolute	6.0 mm	Good	Cu/Microwave
Figure 21d	Linear/Absolute	6.0 mm	Good	Conductive Ink/PEN
Figure 21e	Linear/Absolute	6.0 mm	Good	Conductive Ink/Paper
Figure 26	Linear/Absolute	4.0 mm	Good	Cu/Microwave
Figure 28	Linear/Absolute	4.0 mm	Good	Conductive Ink/Polycarbonate
[17]	Rotary/Incremental	2.25°	Poor	Cu/Microwave
[18]	Rotary/Incremental	0.3°	Poor	Cu/Microwave
[19]	Rotary/Incremental	0.6°	Poor	Cu/Microwave
[21]	Linear/Incremental	0.6 mm	Poor	Cu/Microwave
[23]	Linear/Incremental	0.6 mm	Poor	Cu/Microwave

^1^ The considered robustness is against mechanical wearing or friction. ^2^ Material refers to the one of the inclusions and to the one of the host substrate of the encoder.
